# Therapeutic Implications and Regulations of Protein Post-translational Modifications in Parkinsons Disease

**DOI:** 10.1007/s10571-024-01471-8

**Published:** 2024-07-03

**Authors:** Twinkle Mishra, Shareen Singh, Thakur Gurjeet Singh

**Affiliations:** Chitkara College of Pharmacy, Chitkara University, Punjab, 140401 India

**Keywords:** Parkinsons disease, Post-translational modifications, Protein aggregates, Dopaminergic degeneration, PTM-targeted interventions

## Abstract

Parkinsons disease (PD) is a neurodegenerative disorder characterized by dopaminergic neuron loss and alpha-synuclein aggregation. This comprehensive review examines the intricate role of post-translational modifications (PTMs) in PD pathogenesis, focusing on DNA methylation, histone modifications, phosphorylation, SUMOylation, and ubiquitination. Targeted PTM modulation, particularly in key proteins like Parkin, DJ1, and PINK1, emerges as a promising therapeutic strategy for mitigating dopaminergic degeneration in PD. Dysregulated PTMs significantly contribute to the accumulation of toxic protein aggregates and dopaminergic neuronal dysfunction observed in PD. Targeting PTMs, including epigenetic strategies, addressing aberrant phosphorylation events, and modulating SUMOylation processes, provides potential avenues for intervention. The ubiquitin–proteasome system, governed by enzymes like Parkin and Nedd4, offers potential targets for clearing misfolded proteins and developing disease-modifying interventions. Compounds like ginkgolic acid, SUMO E1 enzyme inhibitors, and natural compounds like Indole-3-carbinol illustrate the feasibility of modulating PTMs for therapeutic purposes in PD. This review underscores the therapeutic potential of PTM-targeted interventions in modulating PD-related pathways, emphasizing the need for further research in this promising area of Parkinsons disease therapeutics.

## Introduction

**Fig. 1:**
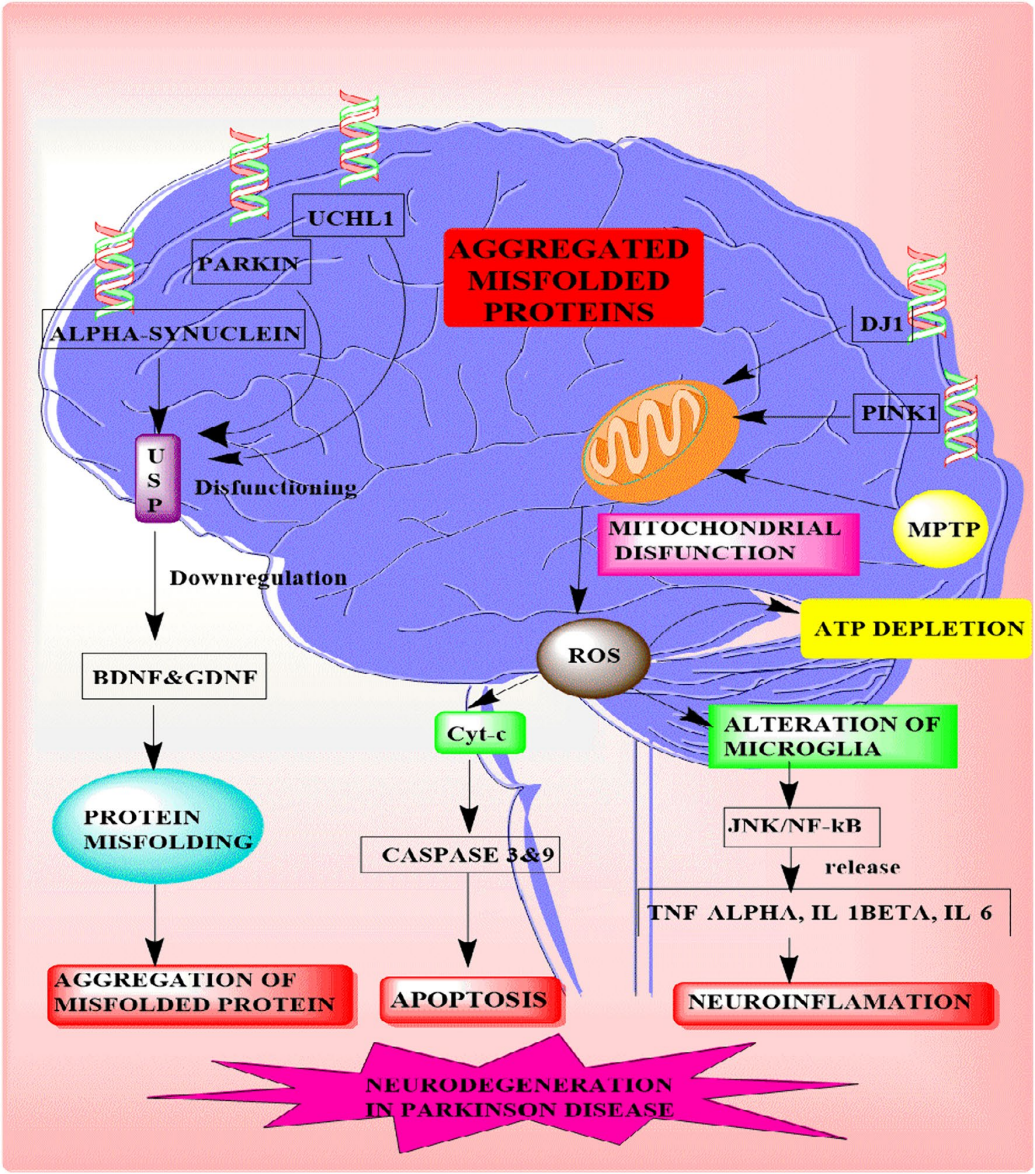
Schematic representation of accumulation of misfolded proteins involved in neurodegeneration in PD

**Fig. 2:**
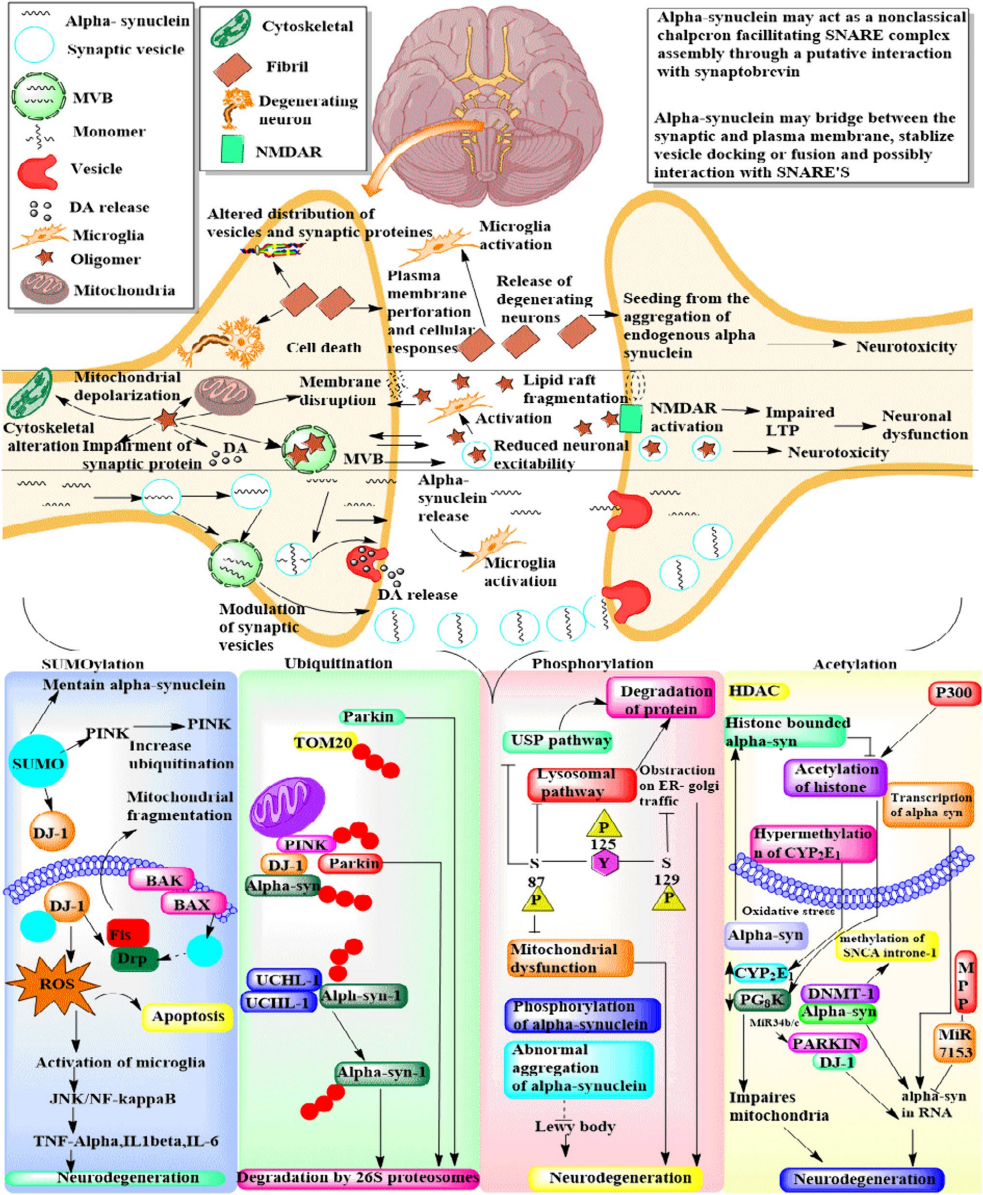
Representation of abnormal pathogenic proteins (Alpha-synuclein, PTEN-induced kinase 1 (PINK1), Parkin, TOM20) confirmations linked involvement of post-transitional modifications directly regulating further intracellular cascades of NB and apoptosis mediated neurodegeneration of PD

Parkinsons disease (PD) is a prevalent neurodegenerative disorder characterized by bradykinesia, resting tremor, rigidity, gait impairment, and postural instability (Jankovic 2008; Sharma et al. 2021a, b). The pathogenesis of PD involves two main hallmarks: the depletion of dopaminergic neurons in the substantia nigra pars compacta region and the accumulation of intracellular aggregates known as Lewy bodies, primarily composed of the alpha-synuclein protein in dopaminergic neurons, leading to their degeneration (Wakabayashi et al. 2013). Despite extensive research into Parkinsons disease (PD), the precise mechanisms underlying dopaminergic neuronal loss and alpha-synuclein aggregation remain unclear (Sidhu et al. 2004). Genetic studies have revealed several genes associated with early onset PD, including UCHL1, alpha-synuclein, DJ1, Parkin, and PINK1, providing crucial insights into pathological conditions (Barcia et al. 2004). Moreover, the compound 6RK73 has demonstrated selectivity in inhibiting UCHL1, an enzyme that cleaves ubiquitin. The protective mechanism of 6RK73 against PD, attributed to its ability to address reduced UCHL1 activity linked to neurodegenerative diseases, presents a novel approach to modulating post-translational modifications (PTMs) (Kooij et al. 2020). Additionally, the postmortem examinations of the substantia nigra pars compacta in PD patients reveal the presence of Lewy bodies and the loss of dopaminergic neurons, confirming the disease (Perry et al. 2008). Disrupted cellular and molecular processes, including diminished lysosomal activity, endoplasmic reticulum stress-related mechanisms, and the occurrence of inflammatory, autophagic, and apoptotic-like cell death events, are linked to the formation of alpha-synuclein fibrillar aggregates (Kalia et al. 2013; Vidyadhara et al. 2019; Maiti et al. 2017).These processes collectively contribute to dopaminergic neurodegeneration in Parkinsons disease (PD) (Zhang et al. 2017). Alpha-synuclein, expressed mainly at presynaptic terminals, is implicated in neurotransmitter storage, segregation, and recycling, affecting neurophysiological functions (Furuta et al. 2010). Aberrations in alpha-synuclein have been linked to age-dependent neurological impairments, affecting synaptic integrity, neurotransmitter release, and vesicle recycling, contributing to neurodegeneration in PD as illustrated in Fig. 1 (Kalia et al. 2013; Limanaqi et al. 2018; Morris et al. 2019). The accumulation of alpha-synuclein has been associated with increased cytotoxicity, inflammatory processes, neuronal apoptosis, mitochondrial dysfunction, and endoplasmic reticulum stress-mediated dopaminergic neurodegeneration in PD (Perry et al. 2008). Moreover, research has focused on the impact of aggregated alpha-synuclein on mitochondrial dysfunction, mitochondrial biogenesis changes, and lysosomal disease-mediated neurodegeneration in PD, as illustrated in Fig. 2. Alpha-synuclein and oligomers disrupt translocation pathways, affecting the translocase outer membrane 20 (TOM20) receptor necessary for mitochondrial protein import (Vartiainen et al. 2006). Additionally, alpha-synuclein blocks the voltage-dependent anion (VDAC) channel on the outer mitochondrial membrane, influencing metabolite outflow and influx in mitochondria. In dopaminergic cell lines, alpha-synuclein oligomers cause mitochondrial fragmentation, calcium-induced inflammation, depolarization, and cytochrome C release, leading to programmed cell death in dopaminergic neurons (Brustovetsky et al. 2002; Rovini et al. 2020; Eriksen et al. 2005; Luth et al. 2014). Furthermore, mutations in genes such as Parkin and PINK1, which are directly linked to mitochondrial malfunctioning, are prevalent in autosomal recessive juvenile parkinsonism (ARJP). Notably, Parkin mutations are associated with impaired ubiquitination and mitochondrial dysfunction, contributing to the pathogenesis of PD (Dawson and Dawson 2010). Parkin, an E3 ubiquitin ligase, is critical in tagging damaged proteins for degradation via the ubiquitin–proteasome system and is crucial for maintaining mitochondrial function. The genetic variations, particularly in DJ1, Parkin, and PINK1, highlight the significance of post-translational modifications (PTMs) in regulating the functions of proteins associated with PD. DJ-1 undergoes reversible oxidative modifications, Parkin is regulated by ubiquitination and phosphorylation, and PINK1 undergoes phosphorylation, all vital for maintaining cellular homeostasis (McNally, 2010; Kahle et al. 2009; Maita et al. 2013). Dysregulation of these PTMs can contribute to cellular dysfunction, providing valuable insights into the development of PD. Thus, the PTMs have been implicated in alpha-synuclein aggregation, further linking these modifications to the pathological causes of PD, especially in the context of mutant genes like Parkin. Cycloheximide, recognized for its influence on protein synthesis, has demonstrated significance in addressing the phosphorylation of PINK-1, a protein associated with early onset familial Parkinsons disease. Therefore, targeting PINK-1 phosphorylation suggests a potential impact on the progression of the PD (Jin and Youle 2012). However, the dysregulation of leucine-rich repeat kinase 2 (LRRK2) phosphorylation is also another key factor linked to aberrant kinase activity, contributing to neurodegeneration in Parkinsons disease (PD) (Nuytemans et al. 2010). Both alpha-synuclein and LRRK2 are frequently implicated in neurological symptoms observed in familial and sporadic PD, with post-translational modifications (PTMs) affecting these proteins commonly detected in Lewy bodies, a pathological hallmark of PD (Singh et al. 2013; Rehni and Singh 2013; Simpson et al. 2020). PTMs, encompassing processes like ubiquitination, phosphorylation, and sumoylation, have emerged as crucial modulators of the pathogenic pathways involving alpha-synuclein and LRRK2 (Singh et al. 2013; Rehni et al. 2010; Simpson et al. 2020). In the context of LRRK2s significance in Parkinsons disease, mutations in the LRRK2 gene are recognized as significant genetic contributors to both familial and sporadic forms of PD (Shyu et al. 2006; Williams and Paulson 2008; Sharma and Singh 2020). LRRK2 is a multidomain protein exhibiting both kinase and GTPase activities, with phosphorylation being a particularly well-studied post-translational modification affecting its functionality (Sharma and Singh 2020). Aberrant phosphorylation of LRRK2 is associated with increased kinase activity and has been implicated in the pathogenesis of PD (Mamais et al. 2018). Understanding the impact of PTMs, particularly phosphorylation, on LRRK2 is essential for unraveling the intricate molecular mechanisms contributing to Parkinsons disease, offering potential insights into therapeutic strategies targeting these specific modifications (Sonustun et al. 2022). Therefore, the current review underlies the intricate relationship between post-transitional modifications, genetic alterations, and the mechanisms of dopaminergic neurodegeneration in Parkinson disease, shedding light on potential therapeutic avenues for this debilitating disorder.

## Post-translational Modifications and Proteinopathies in PD

### SUMOylation in Parkinsons Disease

SUMOylation, a reversible post-translational modification involving the covalent binding of the 12 kDa small ubiquitin-like modifier (SUMO) to lysine residues in target proteins, plays a crucial role in diverse cellular activities. These activities encompass cell cycle regulation, nuclear-cytosolic transport, gene transcription, protein stability, stress response, and apoptosis. Despite its involvement in various cellular functions, SUMOylation primarily targets proteins for degradation. This post-translational modification significantly influences protein structure, stability, localization, and interactions. In neurodegenerative disorders, SUMOylation has emerged as a key player, impacting proteins associated with neurodegeneration, including tau and alpha-synuclein (Dorval and Fraser 2006). The accumulation and aggregation of alpha-synuclein are central features of Parkinsons disease (PD), although the underlying molecular mechanisms remain unclear (Maiti et al. 2017). Several aspects of alpha-synuclein, such as degradation, protein–protein interactions, and subcellular targeting, are regulated by post-translational modifications, with SUMOylation playing a crucial role. Under various stress conditions, there is an observed increase in the SUMOylation of proteins, including alpha-synuclein, which may contribute to neurodegeneration in Parkinsons disease (PD) (Eckermann 2013; Venda et al. 2010; Savyon and Engelender 2020). The alpha-synuclein protein, encoded by soluble NSF attachment protein receptor (SNARE), is implicated in dopaminergic neurodegeneration in PD, as depicted in Figs. 1 and 2. Mutations in the SNARE protein have been associated with alpha-synucleinopathies, playing a role in processes that lead to mitochondrial and lysosomal damage (Venda et al. 2010). Additionally, these mutations contribute to disturbances in the activity of the antioxidant protein DJ-1. DJ-1, known for its antioxidative properties, is subject to regulation through SUMOylation (Moore et al. 2005; Venda et al. 2010; Savyon and Engelender 2020). This regulatory process influences the Nrf2 transcription factor, thereby preventing apoptotic neuronal death. DJ-1 achieves this by inhibiting the SUMOylation of critical proteins such as p53 and BAX (Kahle et al. 2009; Yamane et al. 2015), as illustrated in Figs. 1 and 2. However, mutations in DJ-1, such as the L166P mutation, have been linked to abnormal SUMOylation, suggesting a potential connection between mutated proteins and altered SUMOylation-mediated neurodegeneration in PD (Yamane et al., 2015; Miyazaki and Asanuma 2020). Additionally, the E3 ligase Parkin, belonging to the RBR family (RING), plays a crucial role in cellular protection by destroying misfolded proteins. Its subcellular expression is associated with diverse functions, including gene regulation, mitochondrial quality control, oxidative stress neutralization, and endoplasmic reticulum stress neutralization (Galleguillos et al. 2010; Gatti et al. 2015). Parkin activates ubiquitin to promote monoubiquitination and polyubiquitination of substrates, leading to protein degradation. In the context of Parkinsons disease (PD), the mutated PTEN-induced putative kinase 1 (PINK1) gene is implicated in disrupting the ubiquitin–proteasome system, resulting in the impaired accumulation of proteins and contributing to dopaminergic neurodegeneration in PD (Wilkinson et al. 2010; Bonifatie 2014). The intricate involvement of SUMOylation in such processes, along with the dysregulation observed in PINK1 mutations, highlights the significance of the ubiquitin–proteasome system and SUMOylation as crucial regulatory mechanisms in the complex pathophysiology of Parkinsons disease as illustrated in Fig. 2. The interplay between these molecular processes underscores their collective impact on cellular homeostasis and preventing neurodegenerative processes in PD

### Ubiquitin-Dependent Signaling Pathways in PD

Ubiquitination in Parkinsons disease has become a focal point of investigation with the discovery of nearly 20 genes implicated in rare familial forms of the illness, shedding light on the molecular underpinnings of the condition (Hashimoto and Masliah 1999). These genes, associated with critical cellular processes such as membrane trafficking, autophagy, protein misfolding, and synaptic function, include key components of ubiquitin signaling, namely Parkin, F-box only protein 7 (Fbxo7), PINK1, and the ubiquitin signaling target substrate alpha-synuclein (Hashimoto and Masliah, 1999). The significance of these genes underscores the role of ubiquitination in Parkinsons disease, providing a conceptual framework to understand how mutations in these genes may influence ubiquitylation processes critical for protein quality control pathways (Pereira 2013). Ubiquitination is orchestrated by a series of enzymes, including the Ub-activating enzyme (E1), Ub-conjugating enzyme (E2), and Ub-ligating enzyme (E3). In the context of Parkinsons disease, the identification of alpha-synuclein as a key protein has prompted extensive research into the role of ubiquitination in controlling alpha-synuclein misfolding, as illustrated in Fig. 2. This process plays a pivotal role in the degradation of neuronal pathways and contributes to the downregulation of alpha-synuclein, offering insights into potential therapeutic strategies (Kawakami et al. 2001). Furthermore, alterations in proteasomal subunits and activity, particularly in the substantia nigra of individuals with Parkinsons disease, highlight the intricate involvement of ubiquitination in protein degradation. Chronic systemic treatment with a proteasome inhibitor has been linked to a Parkinsonian phenotype, characterized by dopaminergic neuron loss and the formation of alpha-synuclein-effective inclusions, emphasizing the complex interplay between ubiquitination and the pathogenesis of Parkinsons disease (Davies et al. 2014). These findings underscore the importance of elucidating ubiquitin-mediated pathways for developing targeted therapeutic interventions in Parkinsons disease.

#### Ubiquitination and Parkin in PD

Parkin, a protein associated with Parkinsons disease (PD), plays a crucial role in ubiquitination processes, collaborating with ubiquitin-conjugating enzymes UbcH8 and UbcH7 (E2s). The recruitment of the E2 component by Parkins RING finger domain underscores its involvement in ubiquitin signaling. The ubiquitin-like (UBL) motif in Parkin serves as a proteasome-modifying element, facilitating the transport of multi-ubiquitinated substrates to the ubiquitin–proteasome system (UPS) (Boutell and Sadis 2002). Mutations in the Parkin gene, inherited within families, can impact Parkins binding to UbcH7 and UbcH8 and its E3 ligase activity. Such mutations are implicated in Autosomal Recessive Juvenile Parkinsonism (AR-JP), where the downregulation of Parkins enzymatic activity contributes to the conditions pathophysiology (Imai et al. 2000). Alterations in Parkin function, associated with genetic changes in animal models, have been studied to comprehend better the pathogenic processes underlying Parkinsons disease (Boutell and Sadis 2002). Under dopaminergic neuronal stress, the Parkin-mediated K-63 ubiquitination has been linked to NF-kB signaling, a mechanism newly associated with non-selective mitochondrial macroautophagy (mitophagy) (Sharma et al. 2018). Parkins role in ubiquitination extends to the ubiquitination of mitochondrial proteins in conjunction with alpha-synuclein and PINK-1, facilitating the macroautophagy of damaged mitochondria, as illustrated in Fig. 2. Ubiquitin-like modifiers play a broader role in modulating interactions with specific macroautophagy receptors like p62, highlighting the intricate involvement of Parkin in cellular quality control mechanisms (Kirkin and Dikic 2007). Parkin mutations also influence the regulation of dopamine levels by affecting CDCrel-1, leading to Parkinsonian symptoms. The parkin-associated endothelial-like (Pael) receptor, identified as a second Parkin substrate, is implicated in cellular stress responses and neuronal cell death. Parkins ability to ubiquitinate and down-regulate insoluble parkin-associated endothelial-like (Pael) receptors provides a mechanism for cellular protection under stress conditions. Additionally, Parkin has been shown to protect against proteasomal malfunctioning and toxicity caused by alpha-synuclein, further emphasizing its neuroprotective properties and its potential significance in understanding Parkinsons disease pathology (Betarbet et al. 2005; Qiao et al. 2008; Doyle et al. 2011; Pirooznia et al. 2020). Parkins multifaceted role in ubiquitination and its neuroprotective functions make it a promising candidate for an in-depth investigation into its interactions with the UPS and its involvement in PD pathogenesis.

#### Ubiquitination: Alpha-Synuclein in PD

Ubiquitination processes have emerged as hallmarks of neurodegeneration and are intricately involved in the pathogenesis of Parkinsons disease (PD), particularly through the aggregation of Lewy bodies (LBs) containing immunoreactive alpha-synuclein and ubiquitin proteins (Oueslati et al. 2010). Ubiquitination plays a crucial role in the formation of mono-, di-, and tri-ubiquitinated alpha-synuclein species found in LBs, representing a pathophysiologic hallmark in PD (Oueslati et al. 2010). Notably, the ubiquitination of alpha-synuclein is linked to specific proteins, such as the C-terminal U-box domain of co-chaperone Hsp70-interacting protein (CHIP), seven in absentia homolog (SIAH), and neural precursor cell-expressed, developmentally down-regulated gene 4 (Nedd4) (Sugeno et al. 2014). Within this context, SIAH, identified as a family of RING-type E3 ligases in humans, has been implicated in the ubiquitination of alpha-synuclein, promoting the formation of inclusions (Zhang et al. 2019). In both in vivo and in vitro investigations, SIAH has been found to ubiquitinate alpha-synuclein, leading to the formation of higher molecular weight alpha-synuclein species. Experimental studies using PC12 cells and SH-SY5Y human neuroblastoma cells, along with electron microscopy, have provided insights into SIAH-mediated ubiquitination enhancing alpha-synuclein aggregation and the development of alpha-synuclein-positive inclusions (Muntané et al. 2012; Chorfa et al. 2013; Muntané et al. 2012; Nascimento et al. 2020). Another player in the ubiquitination of alpha-synuclein is the co-chaperone Hsp70-interacting protein (CHIP). This multidomain protein, featuring tetratricopeptide and Hsp70-binding domains, exhibits high-affinity binding with the U-box/ubiquitin ligase. CHIP is known to detect protein misfolding, and its involvement in the ubiquitination of alpha-synuclein has been associated with neuroprotective strategies for Parkinsons disease (Zhang et al. 2019; OHara et al. 2020). Elevated alpha-synuclein levels are observed in PD pathology, disrupting neuronal physiological functions related to neurotransmitter storage and recycling and accumulating Lewy bodies in presynaptic terminals (Zhang et al. 2019; OHara et al. 2020). Cell culturing studies reveal that CHIP is implicated in monoubiquitination and polyubiquitination of alpha-synuclein, co-localizing with Lewy bodies, as illustrated in Fig. 2. This association links these processes to dopaminergic neuron degeneration and the impairment of dopaminergic circuits, contributing to specific behavioral and motor disturbances in Parkinsons patients. (Zucchelli et al. 2010). Notably, compounds like ZPD-2, Leuco-Methylthioninium BIS(Hydromethane sulfonate) (LMTM), NPT200-11, and Ginkgolic Acid exhibit selectivity towards alpha-synuclein, a protein intricately linked to vesicle trafficking in PD (Table 1). These compounds effectively hinder alpha-synuclein aggregation and impede its seeded polymerization, presenting a potential approach to alleviate the formation of toxic protein aggregates (Rott et al. 2017a, b; Peña-Díaz et al. 2019; Schwab et al. 2018; Price et al. 2018). Various research suggested that the administration of ginkgolic acid, an inhibitor of the SUMO E1 enzyme, results in a reduction in α-synuclein levels. Elevated SUMOylation levels are associated with an increased propensity for α-synuclein aggregation and the onset of Parkinsons disease (Eckermann 2013). These findings suggest that interventions targeting SUMOylation activities, including the inhibition of the SUMO E1 enzyme, could be a therapeutic strategy to reduce α-synuclein-related pathology in Parkinsons disease (Dorval and Fraser 2006; Rott et al. 2017a, b; Vijayakumaran et al. 2019). Moreover, the study indicates that inhibiting the proteasome system appears beneficial in Parkinsons disease (PD) (Rott et al. 2017a, b). Dysfunctions in the proteasome system and abnormalities in ubiquitination processes have been implicated in Parkinsons disease (Sun et al. 2007). Compounds such as epoxomicin, PSI, lactacystin (MG132), and SUMO1-15–55 demonstrate the ability to inhibit the proteasome system, leading to a reduction in alpha-synuclein levels and mitigating dopaminergic degeneration in PD (Inden et al. 2005; Sun et al. 2007; Rott et al. 2017a, b). Consequently, targeting ubiquitination and the proteasome degradation system emerges as a potential strategy in preventing Parkinsons disease (PD).

**Table 1:** Drugs targeting proteins linked Post-translational modifications (PTMs) in Parkinsons disease (PD)

S. No.	Drug	Target	Mechanism	References
	ZPD-2, Leuco-Methylthioninium BIS(Hydromethane sulfonate) (LMTM) NPT200-11 Ginkgolic Acid	Alpha-synucleinis an intrinsically disordered protein that is associated with the vesicle trafficking	ZPD, LMTM, NPT200-11 Selectively targets the alpha-synucleinprotein and inhibits the aggregation,and blocks the seeded polymerization of alpha-synuclein	Rott et al. (2017a, b), Peña-Díaz et al. (2019), Schwab et al. (2018), Price et al. (2018)
	Cycloheximide	PINK is mutated in many cases of early onset familial Parkinsons disease	Cycloheximide inhibits the protein synthesis which blocks the PINK-1	Jin and Youle (2012)
	FT3967385	USP30 trigger for Parkin-dependent amplification leading to autophagy death in Parkinson disease	FT3967385 selectively targets the USP30 and prevents the ubiquitylation events that further reduce the Parkin-dependent amplification leading to autophagy in Parkinsons disease	Rusilowicz-Jones et al. (2020)
4	S-Nitrosylation	PARKIN can mediate autophagy of damaged mitochondria, and the malfunctioning of it can lead to Parkinson disease	The S-nitrosylation of PARKIN can decrease the activity like decrease ubiquitination of parkin substance, protein aggregation, and neurodegeneration which further plays a protective role against the PD	Sharma et al. (2021a, b)
5	6RK73	UCHL1 can cleave ubiquitin, and that mutation and reduced activity of this 13 enzyme have been associated with neurodegenerative diseases	6RK73 selectively inhibits UCHL1 in the presence of DUBs in cells. Which plays the protective mechanism against the PD	Kooij et al. (2020)
6	Zebalurine	DNA methylationis an epigenetic mechanism by which methyl groups are added to DNA, playing a crucial role in gene expression regulation	In N2a-APP cells and treatment with zebularine, a DNMT inhibitor lowers the nuclear DNMT1 levels in postmortem PD brain samples which reduces the neuronal death in PD patients	Xu and Li (2012)

#### Ubiquitination: Nedd4 in PD

Nedd4, a HECT-domain E3 ligase enzyme, is crucial in the intracellular ubiquitination process and protective mechanisms associated with Parkinsons disease (PD). Through upregulation, Nedd4 ligase has been shown to decrease the aggregation of alpha-synuclein, a central protein linked to PD pathology. Upon activation, the Nedd4 ligase enzyme facilitates endogenous lysosomal degradation by directly binding to accumulated alpha-synuclein, presenting itself as a potential protective mechanism. Experimental evidence from Drosophila and animal models utilized in Parkinsons research studies suggests that Nedd4-mediated degradation acts protectively against alpha-synuclein-induced toxicity. Drosophila and animal model investigations have unveiled that Nedd4-1-linked Lys-63 ubiquitination influences alpha-synucleins fate, directing its localization to endosomes (Volpicelli-Daley et al. 2014). Studies emphasize the pivotal role of the Nedd4 ortholog Rsp5 in mitigating alpha-synuclein toxicity. Rsp5 aids alpha-synuclein clearance through interactions, ubiquitination, and enhanced destruction mechanisms (Lopes da Fonseca et al. 2015; Alexopoulou et al. 2016). These findings underscore Nedd4-mediated ubiquitinations critical role in alleviating alpha-synuclein-induced neurotoxic effects in Parkinsons disease (PD). The site-specific effects of ubiquitination on alpha-synuclein aggregation and clearance provide essential insights. Specifically, monomeric ubiquitination at K6 emerges as a potent inhibitor of fibril development, offering a protective mechanism against neurodegeneration in PD (Kim et al. 2011). This specificity hinders alpha-synuclein fibril formation and suggests a potential therapeutic avenue for PD intervention. Fibrillar structures typically arise from alpha-synuclein ubiquitination at K10 and K23 (Krumova et al. 2011). Modest inhibition of fibril formation is observed with ubiquitination at K6, K12, and K21. Notably, no fibrils form following ubiquitination at K32, K34, K43, and K96 (Krumova et al. 2011). The integration of K48-linked di- or tetra-ubiquitin chains onto the side chain of Lys12 of alpha-synuclein significantly inhibits fibril formation and regulates clearance, presenting potential therapeutic strategies for managing PD-related alpha-synuclein toxicity (Kim et al. 2011; Zhang et al. 2019). This intricate interplay between ubiquitination and Nedd4-mediated processes suggests promising avenues for PD treatment. Targeting Nedd4-mediated ubiquitination of α-synuclein is a promising therapeutic strategy for mitigating Parkinsons disease and other α-synucleinopathies (Eller and Williams 2011; Altay et al. 2022; Lashuel et al. 2022). This suggests that ubiquitination post-translational modification, which is crucial for alpha-synuclein degradation via the endosomal-lysosomal pathway, holds therapeutic potential. Natural compounds like Indole-3-carbinol (I3C) can modulate Nedd4 ubiquitin ligase activity, preventing alpha-synuclein aggregation and blocking neuroinflammatory NF-kB signaling pathway activation (Cao et al. 2018; Miyazaki and Asanuma 2020; Yong et al. 2023). This targeted approach presents a multifaceted strategy to address neurodegenerative processes linked to alpha-synucleinopathies involved in Parkinsons disease. Also, the findings emphasize the therapeutic potential of targeting Nedd4 to address α-synuclein-associated trafficking defects in Parkinsons disease. A deeper understanding of the molecular intricacies of NAB2-Nedd4 interactions and their downstream effects on ubiquitination provides a foundation for developing targeted interventions. The research suggests avenues for modulating the ubiquitin–proteasome system to alleviate the pathological processes associated with Parkinsons disease (Glickman and Ciechanover 2002). The benzimidazole NAB2 has demonstrated efficacy in rescuing α-synuclein-associated trafficking defects, particularly in the context of early onset Parkinsons disease, and acts in a Nedd4-dependent manner (Hatstat et al. 2021) (Table 1). The potential of Nedd4 modulation offers a novel approach to address the underlying molecular mechanisms contributing to Parkinsons disease, bringing new prospects for developing targeted therapies.

#### Ubiquitination: PD and Autophagy

Parkinsons disease (PD) is associated with disruptions in various cellular pathways, including the proteasomal, lysosomal, and autophagic systems, as indicated by multiple investigations (Oueslati et al. 2010; Cleeter et al. 2013; Jang, 2022). Notably, a reduction in proteasomal activity is observed in the substantia nigra of PD individuals, linking it to a lysosomal storage disorder and increased susceptibility to PD (Jang, 2022). Additionally, several lysosomal storage diseases contribute to autophagic failure, accumulating ubiquitinated protein inclusions (Jang, 2022). The A53T mutation of alpha-synuclein has also been identified as a factor contributing to autophagic cell death (Bernal-Conde et al. 2019). The mechanisms underlying alpha-synuclein degradation are still under debate, particularly regarding the involvement of the proteasomal, chaperone-mediated autophagy, and macroautophagy pathways. Recent research suggests that all three proteolytic pathways are involved in alpha-synuclein breakdown. Notably, inhibiting autophagy proves more effective in impeding alpha-synuclein breakdown than the proteasomal and lysosomal routes, highlighting autophagy as the primary mechanism for alpha-synuclein clearance (Lopes da Fonseca et al. 2015; Xilouri et al. 2016). Alpha-synuclein, implicated in PD, is found in cytosolic inclusions known as Lewy bodies in sporadic forms of the disease **(**Engelender 2008; Sahoo et al. 2022). A fraction of alpha-synuclein from Lewy bodies is monoubiquitinated, and recent research sheds light on the role of this monoubiquitination in Lewy body formation, suggesting a link to the autophagic pathway (Engelender 2008; Sahoo et al. 2022; Jang 2022). The E3 ubiquitin ligase SIAH, present in Lewy bodies, monoubiquitinates alpha-synuclein at lysines associated with Lewy bodies **(**Sahoo et al. 2022; Jang 2022; Altay et al. 2023). Monoubiquitination by SIAH promotes alpha-synuclein aggregation into amorphous aggregates and increases inclusion formation in dopaminergic neurons (Engelender 2008). Autophagy inhibition, and to a lesser extent proteasomal and lysosomal inhibition, promotes the accumulation of monoubiquitinated alpha-synuclein and inclusion formation **(**Sahoo et al. 2022; Jang 2022). These inclusions are toxic to neuronal cells and recruit PD-related proteins, indicating a potential role of monoubiquitination in Lewy body formation. Strategies to decrease alpha-synuclein monoubiquitination, such as preventing SIAH function or stimulating autophagy, could offer new therapeutic avenues for PD (Engelender 2008; Oueslati et al. 2010; Cleeter et al. 2013). Moreover, the involvement of Ubiquitin-specific protease 30 (USP30) in autophagy was validated using FT3967385. Identified as a USP30 trigger for Parkin-dependent amplification leading to autophagy death in Parkinsons disease, FT3967385 selectively acts on USP30. By impeding ubiquitylation events, FT3967385 has the potential to sustain Parkin-dependent amplification, thereby possibly facilitating autophagy in PD (Rusilowicz-Jones et al. 2020) (Table 1).

### Phosphorylation in PD

Phosphorylation, a post-translational modification involving the addition of a phosphate group to proteins, plays a pivotal role in the pathogenesis of Parkinsons disease (PD), a neurodegenerative disorder characterized by the progressive loss of dopaminergic neurons in the substantia nigra region of the brain (Beyer 2006; Prasad et al., 2020). One of the essential proteins implicated in PD is alpha-synuclein, a presynaptic protein that undergoes aberrant phosphorylation at specific serine and tyrosine residues within Lewy bodies, the pathological hallmarks of the disease (Oueslati et al. 2010; Braithwaite and Stock 2012). Notably, phosphorylation of alpha-synuclein at serine 129 (S129-P) is a hallmark of PD, facilitated by kinases such as casein kinase II (CKII) and G protein-coupled receptor kinases (GRK). This phosphorylation event promotes alpha-synuclein fibrillation, oligomerization, and cytoplasmic inclusion formation, potentially contributing to neuronal death and the characteristic motor symptoms of PD (Waxman and Glasson 2011).. In contrast, phosphorylation of alpha-synuclein at tyrosine 125 (Y125) by an unknown kinase has minimal impact on fibrillization, suggesting that different phosphorylation sites may have distinct functional consequences Oueslati et al. 2010). Interestingly, polo-like kinase 2 (PLK2) phosphorylates alpha-synuclein at S129, but rather than promoting pathology, this phosphorylation facilitates the clearance of alpha-synuclein through the lysosomal-autophagic degradation pathway, suggesting a potential neuroprotective role for PLK2 in PD (Alvarez et al. 2010; Chakraborty et al. 2017; Guo et al. 2019). This highlights the complex interplay between kinases and their phosphorylation targets in the context of disease pathogenesis. Another key player in PD is the PINK1 kinase, which regulates the activity and mitochondrial localization of parkin, an E3 ubiquitin ligase implicated in mitochondrial quality control. PINK1-mediated phosphorylation of parkin at specific threonine residues (Thr175/Thr217) is required for its activation and association with the E2 ubiquitin ligase UbcH13/Uev1a, leading to the enhancement of K63-linked polyubiquitination of IKK in the NF-κB signaling pathway (Lim and Lim 2011; Caulfield et al. 2014). Additionally, PINK1-dependent phosphorylation of parkin at serine 65 (Ser65) is crucial for its translocation and stress-induced mitophagy, a process that eliminates damaged mitochondria (Valente et al. 2004; Kondapalli et al. 2012; Caulfield et al. 2014). Mutations in parkin are known to contribute to PD pathogenesis, as they lead to the degradation of Micro1, a parkin substrate involved in maintaining cytosolic calcium levels and promoting mitochondrial clearance. This underscores the potential therapeutic value of targeting the PINK1-parkin pathway through the modulation of phosphorylation events. Beyond alpha-synuclein and parkin, other kinases, such as casein kinase 1 (CK-1), cyclin-dependent kinase 5 (Cdk-5), and c-Abl, also play significant roles in PD through their phosphorylation of various protein targets. For instance, phosphorylation of parkin by CK-1 and Cdk-5 can affect its folding, solubility, and propensity for aggregation, while c-Abl-mediated phosphorylation of parkin at tyrosine 143 can inactivate its E3 ligase activity, highlighting the complex regulatory mechanisms governing parkin function (Shimura et al. 2000; Nuytemans et al. 2010; Chakraborty et al. 2017). Targeting the phosphorylation process represents a promising therapeutic approach for mitigating alpha-synuclein aggregation and regulating kinase activation involved in PD pathology (Chakraborty et al. 2017; Mahul-Mellier et al. 2014). Compounds selectively inhibiting the phosphorylation of kinases, such as CKI, CKII, GRK, LRRK2, and PLK, may regulate alpha-synuclein fibrillization and aggregation, thereby alleviating the toxic effects associated with these events. Modulating the PINK1 pathway through phosphorylation could potentially restore mitochondrial function and protein clearance mechanisms, which are dysregulated in PD (Nuytemans et al. 2010). Notably, certain compounds, including imatinib, nilotinib, bafetinib, and radotinib, have demonstrated efficacy in preventing neurodegeneration in PD by inhibiting tyrosine phosphorylation and suppressing alpha-synuclein aggregation (Werner and Olanow 2022). These findings underscore the potential of targeting phosphorylation as a therapeutic strategy for PD, although further research is needed to fully elucidate the intricate interplay between kinases, phosphorylation events, and disease pathogenesis.

### Methylation and Acetylation Signaling Pathways in PD

DNA methylation constitutes a vital component of the epigenetic machinery, alongside histone modifications and non-coding RNA-mediated gene silencing (Kaidery et al. 2013; Singh et al. 2021; Khan et al. 2021). Within the regulatory elements of the SNCA gene, the methylation of CpG sequences modulates chromatin structure, impeding the access of transcriptional machinery to gene regions, thereby influencing gene expression levels (Mohn and Schubeler 2009). Alterations in methylation status, particularly hypermethylation of promoters associated with gene silencing and demethylation linked to gene activation, significantly contribute to the regulation of gene expression. The pivotal intracellular methylating agent, S-adenosylmethionine (SAM), crucial for this process, is synthesized in neuronal cells through a complex pathway involving vitamin B6, B12, folate, and homocysteine (HCY) in single-carbon metabolism. DNA methyltransferases (DNMTs) play an indispensable role in facilitating the transfer of methyl groups from SAM to cytosine, resulting in the formation of 5-methyl-cytosine. Perturbations in single-carbon metabolism lead to altered gene expression and DNA methylation levels, contributing to the intricate landscape of neurodegeneration (Coppede 2013). The epigenetic regulation of the SNCA gene and alpha-synuclein in Parkinsons disease (PD) is evident, with monoallelic SNCA alleles and the A53T mutation-associated epigenetic silencing involving histone modifications rather than DNA methylation (Yao et al. 2016). The upregulation of wild-type alleles and the suppression of gene expression through the methylation of SNCA intron-1 further underscore the relevance of epigenetic mechanisms in PD development (Coppedè 2012). Epigenetic control of SNCA presence in PD-affected brain regions is highlighted by decreased DNA methylation of SNCA intron-1 in the substantia nigra, putamen, and cortex of sporadic PD patients (Jowaed et al. 2010).

The interaction between alpha-synuclein and DNA methyltransferase 1 (DNMT1) prevents its nuclear localization, leading to reduced DNMT1 levels in the nucleus of dopaminergic neurons. This interaction contributes to hypo DNA methylation involving the upregulation of CpG islands of SNCA, suggesting a potential role for aberrant DNMT1 subcellular localization in epigenetic alterations in the brain (Arand et al. 2012; Miranda-Morales et al. 2017). In PD, differential methylation patterns in the promoters of UCHL-1, MAPT, and ATP13A2 genes have been explored. While the UCHL-1 genes promoter is hypermethylated in malignancies, studies show inconsistent findings regarding UCHL-1 promoter methylation in the cortex region of PD patients (Miranda-Morales et al. 2017). Investigations into the methylation patterns of the MAPT promoter in various PD-associated conditions reveal no significant differences in CpG methylation between control and diseased samples (Behrens et al. 2010). Similarly, no substantial link is established between DNA methylation of ATP13A2 and changes in the substantia nigra in the gene coding for tumor necrosis factor-alpha (TNFA) (Miranda-Morales et al. 2017). Aging-related alterations in subtelomeric methylation in peripheral leukocytes are observed in PD patients, with short telomeres displaying a constant methylation pattern in PD patients and age-related demethylation in controls (Wüllner et al. 2016).

Histone modifications further contribute to the intricate regulatory network in PD. Chromatin states, including heterochromatin and non-condensate chromatin, play pivotal roles in influencing gene expression patterns. Alterations in histone protein conformation, impacting the access of transcriptional machinery to gene promoters, result in either gene silencing or activation (Bryan et al. 1976; Savica et al. 2017). Notably, histone acetylation is inhibited by the interaction of alpha-synuclein with histones, and histone deacetylase inhibitors (HDACIs) demonstrate a neuroprotective role against alpha-synuclein-mediated toxicities (Coppedè 2014). Alpha-synuclein-induced neurotoxicity in the nucleus involves its direct binding to histone H3, inhibiting histone acetylation. Administration of HDACIs mitigates synuclein toxicity by decreasing histone deacetylase activity and increasing histone H3 acetylation, demonstrating their neuroprotective potential in PD (Meyer 2000; Kontopoulos et al. 2006). In the protection of dopaminergic neurons and the facilitation of alpha-synuclein inclusion formation, histone deacetylase 6 (HDAC6) is instrumental in the cytoprotective response of aggresomes, which capture misfolded proteins through autophagy (Miki et al. 2011; Gupta et al. 2020). Addtionally as indicated in the study, a range of Histone deacetylase inhibitors, including valproate, butyrate, phenylbutyrate, nicotinamide, MS-275, and AGK2, were assessed for their HDAC inhibitory properties. The results demonstrated a neuroprotective effect, wherein these inhibitors played a role in preventing dopaminergic degeneration in Parkinsons disease (PD) (Harrison and Dexter 2013).

Dysregulation of microRNAs (miRNAs) further contributes to PD pathology. MiR-133b, exclusively expressed in midbrain dopaminergic neurons, influences their development and function as part of a negative feedback circuit involving the transcription factor Pitx3 (Junn et al. 2009; Miki et al. 2011; Li et al. 2009). Variations in FGF20 translation due to the disruption of miR-433 binding sites are associated with an increased risk of PD (Harraz et al. 2011). Alterations in the levels of miRNAs (miR-10a, -10b, -132, -212, -495) are observed in the brains of early symptomatic alpha-synuclein(A30P) (de Mena et al. 2010; Harraz et al. 2011). Collectively, the intricate interplay of DNA methylation, histone modifications, and miRNA-mediated mechanisms significantly contributes to the epigenetic landscape in PD (Kaidery et al. 2013). Understanding these complex regulatory processes provides valuable insights into the development and progression of PD, opening avenues for potential therapeutic interventions stargeting these epigenetic mechanisms.

Targeting DNA methylation and histone acetylation emerges as a promising therapeutic approach in Parkinsons disease (PD). Epigenetic dysregulation, including altered DNA methylation patterns and histone modifications, plays a pivotal role in PD pathogenesis (Kaidery et al. 2013). Restoring normal DNA methylation, especially in genes like SNCA, UCHL-1, and MAPT, holds the potential to regulate gene expression and mitigate disease-related pathways. Additionally, interventions that modulate histone acetylation, such as histone deacetylase (HDAC) inhibitors, demonstrate neuroprotective effects, indicating their promise as disease-modifying agents. MicroRNA-mediated strategies further offer avenues for targeted intervention (Miñones-Moyano et al. 2011). While challenges and the need for personalized approaches persist, the pursuit of therapies targeting these epigenetic mechanisms represents a transformative direction in PD research. Lastly, the role of DNA methylation in PD has been explored using Zebalurine, a DNMT inhibitor. Treatment with Zebalurine has shown a reduction in nuclear DNMT1 levels in postmortem PD brain samples, suggesting a potential avenue for mitigating neuronal death in PD patients through the modulation of DNA methylation (Xu and Li 2012;Cheng et al. 2015). In conclusion, these findings collectively underscore the potential of targeting PTMs as a multifaceted approach in managing PD.

### Protein Palmitoylation and De-palmitoylation in PD: Role of Dopamine Receptors

Palmitoylation, a reversible lipid modification involving the attachment of palmitate groups to cysteine residues, plays a crucial role in the regulation of dopamine receptors, which are integral in governing various physiological processes such as executive function, learning, reward, and motivation. Disruptions in dopaminergic signaling have been implicated in neurological disorders, including Parkinsons disease (PD) (Rasheed and Alghasham 2012). Palmitoylation of dopamine receptors, particularly the D1 and D2 receptor families, influences receptor stability, trafficking, and functional outcomes. For instance, palmitoylation of the D1 receptor has been associated with enhanced palmitate integration and agonist-dependent receptor internalization (Hasbi et al. 2010). Additionally, both short and long isoforms of the D2 receptor undergo palmitoylation, impacting receptor stability and cell membrane trafficking. Notably, the D3 receptor exhibits extensive palmitoylation, and this post-translational modification influences PKC-mediated endocytosis and agonist-induced receptor tolerance (Tirotta et al. 2008; Chien et al. 2010). Given the relevance of dopamine receptors, particularly D3 and D2 receptors, in the context of frequently used neuroleptic drugs and their association with motor dysfunctioning, understanding the intricate processes of palmitoylation and depalmitoylation may offer insights into the pathophysiology and potential therapeutic strategies for PD (Hasbi et al. 2010). Further exploration of the role of palmitoylation in D4 receptors adds to the complexity of these regulatory mechanisms in dopaminergic signaling, providing potential avenues for targeted interventions in neurodegenerative disorders like PD (Zhang and Kim 2016; Ebersole et al. 2015; Rankin et al. 2010). Therefore, this suggests that targeting palmitoylation and depalmitoylation processes presents a promising avenue for treatment. Modulating these lipid modifications could potentially restore the balance of dopaminergic receptor function, mitigating the pathological effects observed in PD. Strategies aimed at controlling the palmitoylation status of dopamine receptors may involve the development of pharmacological agents that specifically target the enzymes responsible for these modifications, ensuring precise regulation of receptor activity. As recommended, the administration of palmostatin B (PSB), along with ML348—an APT1-specific inhibitor—and ML349, was investigated for their potential in treating Parkinsons disease (PD). The approach involved enhancing palmitoylation, resulting in the amelioration of various aspects of αS cytopathology. This included improvements in vesicle- and αS-rich inclusions, normalization of abnormal αS phosphorylation, and a reduction in neurotoxicity (Ho et al. 2021).

## Conclusion

In conclusion, post-translational modifications (PTMs), such as DNA methylation, histone modifications, phosphorylation, SUMOylation, and ubiquitination, are integral contributors to the pathophysiology of Parkinsons disease (PD). This review highlights the significant impact of these PTMs on key proteins like alpha-synuclein, tau, and DJ-1, influencing their stability, interactions, and clearance mechanisms. Understanding the functions and regulations of proteins central to PD pathogenesis remains a key research focus. Ongoing efforts to identify specific PTM sites on PD-related proteins and unravel their physiological consequences are expected to advance our understanding of protein functions and the complex control mechanisms. Moreover, determining how these modifications are disrupted in PD sheds a light on potential therapeutic strategies. Dysregulation in such PTMs processes leads to the accumulation of toxic protein aggregates, dopaminergic neuronal dysfunction, and the progressive neurodegeneration observed in PD. Understanding the dynamic interplay of these post-translational modifications sheds light on potential therapeutic targets for PD. Targeting PTMs emerges as a promising therapeutic avenue for managing PD. Epigenetic interventions, such as restoring normal DNA methylation patterns and modulating histone acetylation, hold potential for regulating gene expression and mitigating disease-related pathways. Strategies that address aberrant phosphorylation events associated with alpha-synuclein and PINK1, as well as interventions in SUMOylation processes, present opportunities to alleviate protein aggregation and enhance neuronal protection. The ubiquitin–proteasome system, governed by enzymes like Parkin and Nedd4, offers potential targets for clearing misfolded proteins, contributing to the development of disease-modifying interventions. Compounds like ginkgolic acid and specific inhibitors of SUMO E1 enzyme, as well as natural compounds like Indole-3-carbinol (I3C), demonstrate the feasibility of modulating PTMs for therapeutic purposes.

## Data Availability

Not applicable.

## Abbreviations

ARJP: Autosomal recessive juvenile parkinsonism
PTMs: Post-translational modifications
UCH-L1: Ubiquitin C-terminal hydrolase L1
DJ-1: Dopamine receptor
SUMO: Small ubiquitin-like modifier
PINK1: PTEN-induced kinase1,
PTEN: Phosphatase and tensin homologue-induced kinase 1
Nedd4: Neuronally expressed developmentally downregulated 4
TOM20: Translocase outer membrane 20
SNARE: Soluble NSF attachment protein receptor
NF-kB: Nuclear factor kappa B
Nrf2: Nuclear factor erythroid 2–related factor 2
